# Development of VEP-based biomarkers to assess plasticity states

**DOI:** 10.1038/s41398-025-03676-x

**Published:** 2025-10-20

**Authors:** Viktoria Galuba, Theresa Wolf, Anouk Ihle, Franziska von der Decken, Felix Mülsch, Bernd Feige, Andreas Vlachos, Katharina Domschke, Claus Normann, Stefan Vestring

**Affiliations:** 1 Department of Psychiatry and Psychotherapy, Medical Center, University of Freiburg, Faculty of Medicine, University of Freiburg, Freiburg, Germany; 2 Department of Neuroanatomy, Institute of Anatomy and Cell Biology, Faculty of Medicine, University of Freiburg, Freiburg, Germany; 3 German Center for Mental Health (DZPG), Berlin/Potsdam, Germany

**Keywords:** Diagnostic markers, Psychiatric disorders

## Abstract

Disturbances in neuroplasticity are associated with many psychiatric and neurological disorders. Noninvasive electroencephalography (EEG) recordings of visually evoked potentials (VEPs) are promising for assessing plasticity in the human visual cortex, which may represent long-term potentiation (LTP). However, the variability in stimulation parameters limits the comparability and identification of optimal plasticity-inducing protocols. In this study, we systematically compared four VEP modulation protocols—low-frequency, repeated low-frequency, high-frequency, and theta-pulse stimulation—and assessed their effects on visual cortical plasticity. We analyzed 152 EEG recordings, where VEPs were evoked via a checkerboard reversal stimulus before and after low-frequency, repeated low-frequency, high-frequency, and theta-pulse stimulation. Changes in VEP amplitudes were measured from baseline to 2–28 min postmodulation. Low-frequency stimulation produced transient changes in plasticity, peaking at 2 min but dissipating within 12 min. Repeated low-frequency stimulation induced more sustained changes in plasticity, persisting for up to 22 min. High-frequency stimulation induced sharp but brief increases in plasticity indices, whereas theta-pulse stimulation was associated with moderate but prolonged changes in plasticity, lasting up to 28 min. These findings highlight the crucial influence of stimulation parameters on short- and long-term synaptic plasticity indices. Depending on the objective, a suitable induction protocol can be selected to optimize the desired effects, such as increasing sensitivity to drug effects or targeting longer-lasting plasticity outcomes. Optimized VEP paradigms have strong translational potential for assessing neuroplasticity deficits in individuals with psychiatric and neurodegenerative disorders, paving the way for the development of new biomarkers and therapeutic strategies.

## Introduction

Alterations in synaptic plasticity are associated with the pathophysiology of various psychiatric and neurological disorders [1]. Synaptic plasticity refers to adaptations in the strength or efficiency of synaptic transmission in response to neural activity [2]. Although these changes vary in terms of form and function, they can generally be categorized as either short- or long-lasting and as either enhancing or depressing synaptic transmission [3, 4]. Long-term potentiation (LTP) and long-term depression (LTD) are key mechanisms that strengthen or weaken these connections, respectively, forming the foundation for learning and memory [5]. These mechanisms have been widely investigated through ex vivo studies with brain slices from rodents. However, direct assessment of the correlates of synaptic plasticity in living humans has been challenging [6].

Noninvasive electroencephalographic (EEG) recordings have enabled assessments of synaptic plasticity in humans through investigations of the stimulus-selective response plasticity (SRP) induced by sensory stimulation in the cerebral cortex [7–10]. In a typical experimental protocol, baseline sensory-evoked potentials are recorded, followed by a phase of intense sensory stimulation (modulation phase). Then, changes in the baseline stimulus are assessed to quantify plastic effects over time, providing a measure for SRP. These SRP protocols generally resemble canonical LTP protocols in brain slice experiments: the amplitude of excitatory postsynaptic potentials (EPSPs) is measured, followed by the application of intense stimulation to the same pathway. The resulting poststimulation modulation of EPSP amplitudes is regarded as a measure of LTP. Compared with LTP experiments with brain slices, SRP experiments involve longer and more complex synaptic pathways.

Recordings of visually evoked potentials (VEPs), which were initially demonstrated in rodents [11, 12] and later validated in humans [13, 14], have revealed that prolonged 2- or 9-Hz visual stimulation increases the amplitudes of subsequent VEPs. The VEP paradigm has key Hebbian properties, including N-methyl-D-aspartate receptor (NMDAR) dependency [15], input specificity [16–18], and persistence [11]. These findings provide compelling evidence that SRP-induced VEP changes can serve as indices of LTP-like plasticity in the human primary visual cortex [8]. Additionally, previous research has suggested a coordinated interplay between the plasticity of the visual cortex and hippocampus, a key region associated with learning and memory, during sensory processing [19, 20]. Modulation of VEPs has been shown to be positively associated with human memory performance [21].

However, variations in VEP paradigms related to the stimulus presentation and modulation frequency introduce heterogeneity in the extent and duration of VEP-related plasticity [8, 22]. A critical difference among SRP paradigms is the modulation phase. Two major variations have been developed. In the first paradigm, short (2 min), high-frequency tetanic (∼9 Hz) modulation of a flash stimulus is used [13]. In the second paradigm, a sustained (10 min), slower (2 Hz) pattern reversal modulation technique is applied [14]. Both methods have been shown to elicit LTP-like plasticity in distinct regions associated with early VEP responses [8].

The use of brain slices provides a more controlled setting to examine the effects of variations in the modulation phase on subsequent plastic responses and their underlying mechanisms. In these settings, the frequency and duration of stimulation are key determinants of synaptic plasticity outcomes. High-frequency stimulation (10–200 Hz) not only induces LTP by strengthening synaptic connections but also enables short-term potentiation (STP) phenomena such as post-tetanic potentiation (PTP) [23]. These short-term increases in neurotransmitter release, lasting seconds to minutes, are largely driven by calcium accumulation in the presynaptic terminal during high-frequency stimulation. This residual calcium interacts with subsequent action potentials to either directly increase transmitter release or trigger biochemical modifications in presynaptic proteins [23, 24].

In brain slice LTP experiments, the direction and magnitude of the plastic response critically depend on the frequency and pattern of the synaptic stimulation applied in the modulation phase. Low-frequency stimulation (1–3 Hz) has been shown to induce LTD, which is characterized by decreased synaptic strength through mechanisms distinct from those associated with LTP [25, 26]. Pulsed application of synaptic stimulation, particularly at a theta frequency, has been shown to increase the subsequent potentiation effects. Theta bursts mimic naturally occurring firing patterns in the brain and provide optimal calcium dynamics and synaptic recovery during intervals [27]. In contrast, the extent of potentiation is limited by a ceiling effect when intense high-frequency protocols are applied repeatedly, most likely due to a saturation of synaptic mechanisms or a limitation of calcium dynamics.

Numerous studies have reported impaired VEP-related plasticity in individuals with neuropsychiatric disorders, including major depression disorder [14, 28], bipolar disorder [29, 30], and schizophrenia [29, 31–33], as well as among healthy older individuals [34]. Moreover, antidepressant interventions, such as the use of selective serotonin reuptake inhibitors [14], ketamine [35] and transcranial direct current stimulation [36], have been shown to increase VEP-related plasticity measures. These findings provide translational validation of the results obtained from animal models, suggesting the crucial role of deficits in neuroplasticity in psychiatric disorders. However, in these studies, only significant differences on a group level were described.

Given the need for systematic investigations of modulation parameters [22], we aimed to examine how different stimulation frequencies and patterns affect plastic VEP changes in the human visual cortex. Using EEG-based VEP measurements, we aimed to identify stimulation parameters that induce optimal LTP-like plasticity. While differences in stimulus types (e.g., pattern reversal vs. flash stimulation) are beyond the scope of this study, this approach will help resolve ambiguities associated with variabilities among previous experimental paradigms [7, 8, 21, 22]. Understanding the frequency- and pattern-specific dynamics of plastic effects in the visual cortex will increase the translational relevance of VEP-based paradigms for investigating plasticity deficits in individuals with psychiatric disorders. We seek to optimize VEP-based plasticity paradigms for future use as biomarkers for neuropsychiatric disorders involving neuroplasticity dysregulation and to better evaluate interventions aimed at increasing plasticity, such as pharmacotherapy and noninvasive brain stimulation.

## Methods

### Study design and samples

The Ethics Committee of the Albert-Ludwigs-University Freiburg (approval numbers: 24–1237-S1 and 21–1113) approved all the experiments and the trial was registered in the German Clinical Trials Register (DRKS00034184). All participants provided written informed consent and had no history of neurological, psychiatric, or ocular disorders; were not taking psychoactive medications or illegal drugs; and had normal or corrected-to-normal vision.

**Fig. 1:**
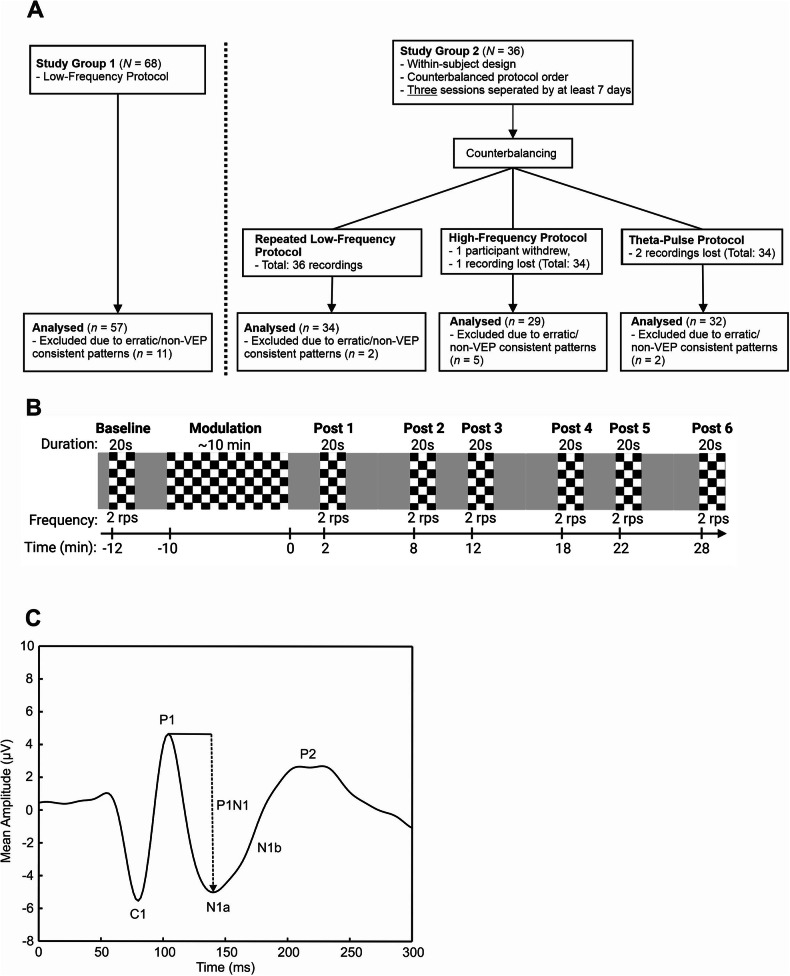
Overview of the study procedures. **A** Study flowchart; **B** Schematic presentation of the VEP-related plasticity paradigm, including the block titles, duration, presentation frequency and time of block onset. The duration of the modulation blocks differed among the protocols. **C** Schematic representation of a pattern reversal VEP and its components.

We report the results of two experimental studies with healthy controls (Fig. 1A). The first study group consisted of 68 participants (42 females, 26 males), with a mean age of 28.8 ± 8.9 years. The VEP recordings were obtained using a low-frequency protocol adapted from Normann et al. [14].

The second study group included 36 participants (23 females, 13 males), with a mean age of 27.08 ± 4.01 years. This group participated in experiments with a within-subject design that involved three protocols—repeated low-frequency, high-frequency, and theta-pulse modulation—applied in a counterbalanced order. Sessions were separated by at least seven days to prevent carry-over effects and were conducted at the same time of day to control for wakefulness and putative effects of the circadian rhythm [37, 38]. Due to the nature of the study, neither participants nor experimenters were blinded during trial conduction. In this exploratory study, a priori orientative power calculations assuming an effect size of *d* = 0.5, a two-sided alpha of 0.05, and a cross-over design indicated that a group size of at least 34 participants would yield a power of ≥80%. A comparison of the age and sex distributions revealed no significant differences between the study groups (Supplementary Table S1).

### Experimental setup and paradigms

The participants were comfortably seated at a distance of 1.71 meters from a 55” OLED screen (LG, model code: OLED55CX9LA) with a refresh rate of 120 Hz. A checkerboard reversal stimulus, adapted from that used by Normann et al. [14], was used to evoke VEPs, with individual checkers subtending a visual angle of 0.5°. All stimuli were generated using Expyriment [39] in Python, and a gray screen was displayed during the intervals between checkerboard stimulation blocks. To maintain attention throughout the recording session [40], the participants were instructed to fixate on a central cross (Ø0.61 cm) and read numbers that appeared randomly within the cross out loud.

Baseline and postmodulation VEPs were recorded by inverting the checkerboard stimulus for 20 s at a temporal frequency of two reversals per second (rps), resulting in 40 sweeps per block. Six postmodulation blocks were recorded at 2, 8, 12, 18, 22, and 28 minu (Fig. 1B).

**Fig. 2:**
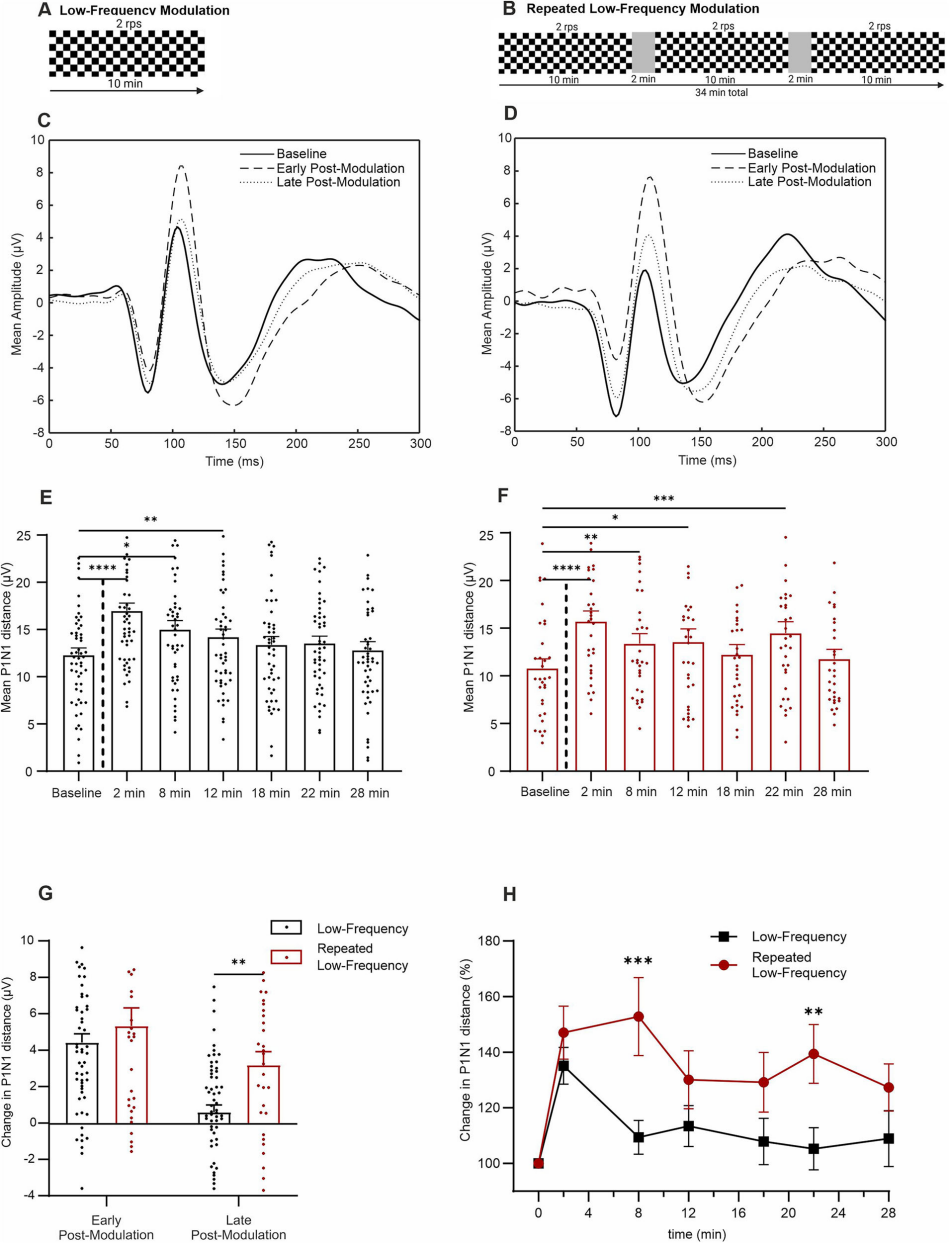
Comparison of the effects of the low-frequency and repeated low-frequency modulation protocols. **A** and **B** Schematic representations of the experimental timeline for each modulation phase; **C** and **D** Grand average VEP traces at baseline, in the early postmodulation period (2 min) and in the late postmodulation period (Ø 22 and 28 min) for the low-frequency (*n* = 57) and repeated low-frequency (*n* = 34) protocols; **E** Mean P1N1 was significantly changed by the low-frequency modulation from baseline to 2 min (*p* < 0.0001), 8 min (*p* = 0.014) and 12 min postmodulation (*p* = 0.0013); **F** Mean P1N1 was significantly changed by the repeated low-frequency modulation from baseline to 2 min (*p* < 0.0001), 8 min (*p* = 0.0075), 12 min (*p* = 0.0465) and 22 min postmodulation (*p* = 0.0005); **G** Mean P1N1 change (baseline value subtracted from postmodulation value) was significantly greater during late postmodulation phases (Ø 22 and 28 min) using the repeated low-frequency modulation protocol (*p* = 0.0011); **H** Post hoc comparison of baseline corrected changes (%) between protocols revealed a significantly greater percentage change after repeated low-frequency modulation at 8 min (*p* < 0.0001) and 22 min postmodulation (*p* = 0.0054). The data are presented as the means ± SEMs. *p < 0.05, **p < 0.01, ***p < 0.001, ****p < 0.0001.

**Fig. 3:**
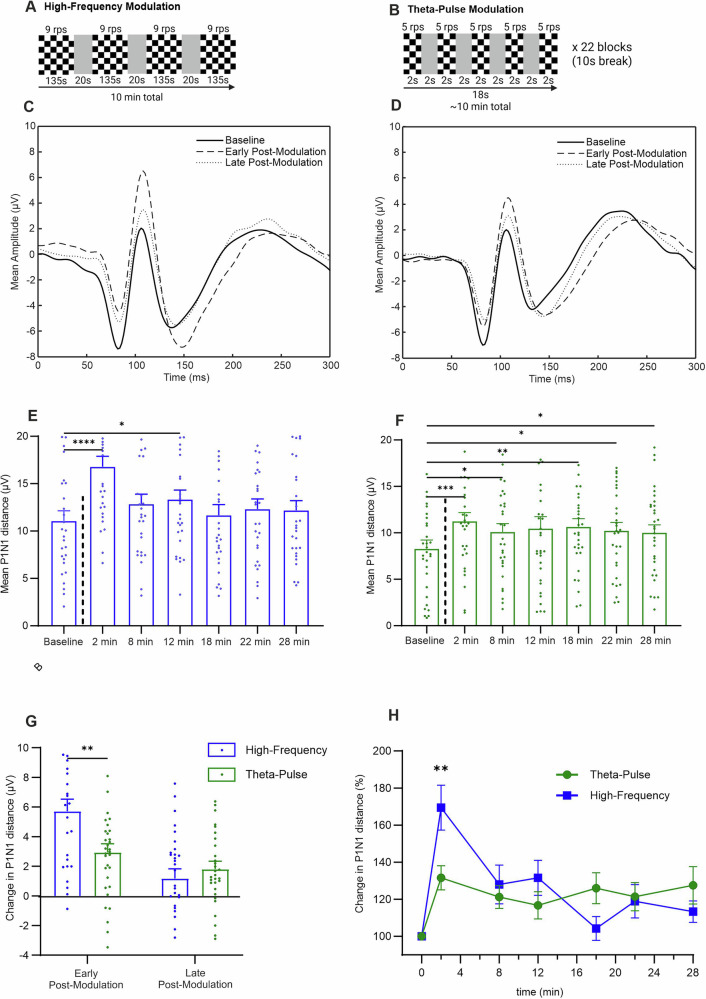
Comparison of the effects of the high-frequency and theta-pulse modulation protocols. **A** and **B** Schematic representation of the experimental procedure for the individual modulation phases; **C** and **D** Grand average VEP traces at baseline, in the early postmodulation period (2 min) and in the late postmodulation period (Ø 22 and 28 min) for the high-frequency (*n* = 29) and theta-pulse stimulation (*n* = 32) protocols; **E** Mean P1N1 was significantly changed by the high-frequency modulation from baseline to 2 min (*p* < 0.0001) and 12 min postmodulation (*p* = 0.0215); **F** Mean P1N1 was significantly changed by the theta-pulse modulation from baseline to 2 min (*p* = 0.0002), 8 min (*p* = 0.0369), 18 min (*p* = 0.0054), 22 min (*p* = 0.0228), and 28 min postmodulation (*p* = 0.0373); **G** Mean P1N1 modulation (baseline value subtracted from postmodulation value) significantly differed between protocols in the early postmodulation period (2 min; *p* = 0.0083). Mean P1N1 modulation was not significantly different in the late postmodulation period (Ø 22 and 28 min; *p* = 0.5298). **H** Post hoc comparison of baseline corrected changes (%) between protocols revealed a significantly greater percentage change after repeated high-frequency modulation at 2 min postmodulation (*p* = 0.0129). The data are presented as the means ± SEMs. *p < 0.05, **p < 0.01, ***p < 0.001, ****p < 0.0001.

The modulation blocks varied depending on the protocol used in the study. The low-frequency modulation protocol consisted of a single 10 min block at a reversal frequency of 2 rps, producing 1200 stimuli (Fig. 2A). We used this protocol as a standard to assess alterations caused by variations in the modulation block in the following experiments. The repeated low-frequency modulation protocol was designed to elicit a putative ceiling effect of potentiation. Here, three 10 min blocks at a reversal frequency at two reversals per second were used, resulting in the application of a total of 3600 stimuli (Fig. 2B). Two other protocols were developed to examine the effects of higher frequency stimulation in a pulsed application strategy. In the high-frequency modulation protocol, four blocks were applied at a reversal frequency of 9 rps, with each block lasting 135 s (Fig. 3A). This resulted in a total modulation phase of 10 min and 4860 reversal presentations. Finally, the theta-pulse modulation protocol included 22 blocks, with each block composed of five segments presented at a frequency of 5 rps, resulting in a total of 1100 stimuli presented over a period of approximately 10 min (Fig. 3B).

### Data acquisition

Continuous EEG data were acquired using *Neuroscan Scan 4.3* (Compumedics Neuroscan). Eight electrodes were positioned according to the international 10–20 system (Supplementary Fig. S1). The reference electrode was placed centrally at Cz, whereas the ground electrode was positioned at Fz. To monitor and account for artifacts caused by eye movements, additional electrodes were placed at AF7, FPz, and AF8. Prior to the recordings, the electrode impedance was reduced to less than 10 kΩ to ensure data quality. VEP signals were recorded via the Oz electrode, which was located 10% above the inion along the mid-sagittal plane. The EEG data were sampled at 500 Hz, and a bandpass filter of 0.1–100 Hz was applied during data acquisition. The event markers were transmitted through a parallel port for precise synchronization.

### EEG preprocessing

The EEG data were preprocessed using EEGLAB (version 2023.1) in a MATLAB environment (R2020b). The custom MATLAB code used to generate the results and perform the analyses presented in this study is available upon qualified request from the corresponding author. A latency correction of 20 milliseconds was applied to align the event markers with the stimulus presentation, compensating for delays introduced by the hardware setup. To reduce noise and remove unwanted frequencies, an offline bandpass filter between 40 Hz and 0.5 Hz was applied. Epochs were segmented using a time window of −100–450 milliseconds relative to the stimulus onset. Baseline correction was applied using the average signal amplitude in the prestimulus interval from −100–0 milliseconds as the reference period. For each epoch, the mean signal amplitude within this time window was calculated and subtracted from all time points, ensuring that the baseline activity was centered at zero and that potential offsets and slow drifts were removed. Epochs in which the threshold exceeded ± 100 µV were rejected. VEP signals were averaged per individual for each block (baseline and postmodulation blocks 1–6).

To ensure data quality, a trainer who was blinded to the experimental conditions visually inspected the averaged VEP block signals. Among the 172 EEG recordings, 20 (11.63%) were deemed insufficient measurements dominated by noise (low-frequency protocol: 11; repeated low-frequency protocol: 3; theta pulse protocol: 2; high-frequency protocol: 5). These recordings were identified on the basis of erratic patterns and patterns inconsistent with VEPs and were manually excluded from further analyses (Supplementary Fig. S2). A chi-square test revealed no statistically significant differences in data exclusion rates among the four protocols (*p* = 0.5556), indicating that the excluded data were evenly distributed across the protocols and that the likelihood of bias was minimized.

VEP components and latencies were automatically extracted using a custom MATLAB script using the following definitions (Fig. 1C): C1 was defined as the most negative value occurring between 50 and 100 milliseconds after stimulus onset, whereas P1 was identified as the most positive value occurring within 70–130 milliseconds after flipping. N1a was defined as the first negative peak following P1, and N1b was calculated as the average activity within 150–190 milliseconds after reversal. P2 was determined as the average activity between 225 and 280 milliseconds after stimulus onset. The difference between P1 and N1 (in µV) was subsequently calculated.

### Statistical analyses

All data analyses were conducted using GraphPad Prism (version 10.0.0; San Diego, California). The investigator performing the statistical analysis was not blinded to the experimental protocols. A *p* value of 0.05 was considered statistically significant, and VEP components are reported as grand averages ± standard errors of the means (SEMs). The P1N1 difference served as the primary outcome; this metric, which was calculated considering changes in two well-characterized VEP components [8] (P1 and N1) for increased sensitivity, is a comprehensive representation of cortical processing. P1N1 has been shown to be a reliable indicator for distinguishing healthy individuals from those with psychiatric disorders [30, 41]. Given that previous work has shown the modulation of other VEP components [42], additional results are provided in the Supplementary Tables S2-S6.

Prior to analyzing the VEP components, statistical outliers within the data were identified and removed using the ROUT method (Q = 1%). With this procedure, we identified and excluded 13 outlying P1N1 data points.

Plasticity-like modulation within each protocol was evaluated using a repeated-measures ANOVA to analyze changes in P1N1 from baseline to 2 to 28 min postmodulation. P1N1 values were tested for normality using Kolmogorov-Smirnov test. When the assumption of sphericity was violated, Greenhouse-Geisser correction was applied. If values were missing, a mixed-effects model with restricted maximum likelihood estimation (REML) was computed. Post hoc *t* tests with Dunnett’s correction for multiple comparisons were conducted to identify specific differences.

To assess between-protocol differences in early (2 min) and late (Ø 22 and 28 min) P1N1 modulation periods, baseline values were subtracted from the values obtained in the respective postmodulation blocks. Unpaired *t* tests were performed to evaluate significant differences.

For a detailed temporal analysis of the modulation effects, individual postmodulation P1N1 values were normalized to their respective baseline values and are expressed as percentage changes. Two-way ANOVA (2 × 6 design) was conducted, with protocol and time as factors and the P1N1 percentage change as the outcome. When significant main effects were found, post hoc t tests with Šídák correction for multiple comparisons were applied to identify specific differences across time points and between protocols.

The temporal stability of the early (2 min) and late (Ø 22 and 28 min) P1N1 modulation periods was assessed for each protocol using Pearson correlations. Modulation between protocols and consistency (ΔEarly-Late Modulation) was calculated as the difference between the values in the early and late modulation periods, reflecting the stability of this plasticity over time. Higher values indicate greater divergence, whereas values near zero suggest greater consistency. One-way ANOVA was conducted, followed by post hoc *t* tests with Tukey’s correction for multiple comparisons to identify specific differences.

Finally, to test whether the pattern-reversal elicited by checkerboards with upper-left corner black (trigger type 1) versus white (trigger type 2) were equivalent, we applied a Two One-Sided Tests (TOST) procedure with a predefined equivalence margin (SESOI) of ± 0.5 µV to assess whether differences in P1N1 amplitude between trigger types 1 and 2 were statistically negligible (Supplementary Fig. S3).

## Results

### Low-frequency modulation induces transient changes

The grand average VEP signals recorded during the application of the low-frequency protocol in the baseline, early (2 min), and late postmodulation (Ø 22 and 28 min) phases are depicted in Fig. 2C. The analysis of the effect of low-frequency modulation on P1N1 included the data of 57 participants, and P1N1 values were compared across seven time points: the baseline and six postmodulation time points (2–28 min). The variance across time was statistically significant (*F*(4.399, 217.0) = 23.29, *p* < 0.0001), indicating robust differences among time points. Post hoc analysis revealed a significant increase in P1N1 values compared with those at baseline at several time points: 2 min postmodulation (mean difference = 4.544, *p* < 0.0001), with the increase in the P1N1 value persisting in the 8 min (mean difference = −1.820, *p* = 0.0122) and 12 min postmodulation periods (mean difference = −2.029, *p* = 0.0011). No significant differences in the P1N1 values compared with those at baseline were observed for later time points, suggesting that the low-frequency protocol induces a significant short-term increase in P1N1, particularly during the immediate and early postintervention phases, with the effect diminishing between 12 and 18 min poststimulation.

Since the first demonstration of visually induced long-term potentiation-like effects using checkerboard stimulation by Kirk et al. and its methodological synthesis in their review, it has been debated whether pattern-reversal VEPs reflect plasticity at the full nominal reversal rate or effectively at half that rate due to alternating contrast polarity [10, 13]. More recent evidence suggests that each contrast change reliably elicits a cortical response at the full nominal frequency [43, 44]. To examine this directly, we analyzed VEPs elicited by transitions to black and to white separately within the low-frequency protocol. As shown in Supplementary Fig. S3, the two transitions produced virtually identical grand average VEPs in amplitude, latency, and topography, supporting the interpretation that each contrast change independently evokes a response at the full reversal frequency. The mean P1N1 amplitudes were highly similar (Type 1:10.91 ± 1.01 µV; Type 2:10.86 ± 1.05 µV; mean paired difference: 0.05 µV). Equivalence testing using the TOST procedure further confirmed statistical equivalence within the predefined margin of ± 0.5 µV (p-lower = 0.0005, p-upper = 0.0033; see Supplementary Fig. S3C).

### Repeated low-frequency modulation induces sustained effects

The VEP signals obtained during the application of the repeated low-frequency protocol, shown in Fig. 2D, demonstrate changes in the P1N1 value in the early (2 min) and late postmodulation phases (Ø 22 and 28 min) compared to the value at baseline. The variance in the mean P1N1 values over time was statistically significant (*F*(3.973,121.2) = 1 1.65, *p* < 0.0001), and post hoc analysis revealed a significant increase in the P1N1 value at 2 min (2 min: mean difference = −5.331, *p* < 0.0001), 8 min (mean difference = −3.078, *p* = 0.0075), 12 min (mean difference = −2.696, *p* = 0.0465), and 22 min postmodulation (mean difference = −3.778, *p* = 0.0005). The reductions in the P1N1 value at 18 min (*p* = 0.0580) and 28 min (*p* = 0.0920) postmodulation were not statistically significant. These results indicate that the repeated low-frequency modulation protocol induced a significant and sustained increase in P1N1, which persisted until the time point typically associated with LTP (>20 min). Although numeric increases in P1N1 were observed at all time points, the effect gradually decreased, with significance differences from baseline observed only in the earlier phases of the observation period (Fig. 2F).

### Low-frequency vs. repeated low-frequency modulation

To compare the effects of the low-frequency and repeated low-frequency protocols, the baseline P1N1 value was subtracted from the value obtained at 2 min postmodulation (early postmodulation effect) and the average P1N1 value obtained at 22 and 28 min postmodulation (late postmodulation effect) for each individual (Fig. 2G). Unpaired t tests did not reveal any significant differences in the early postmodulation phase (*t*(88) = 0.9368, *p* = 0.3514). In contrast, a significant difference was found between the protocols when late modulation effects were analyzed (*t*(90) = 3.374, *p* = 0.0011). These results suggest that the low-frequency and repeated low-frequency protocols do not significantly differ in terms of early modulation effects. However, a significant difference in their effects is observed at later time points (22 and 28 min postmodulation). The findings highlight a protocol-specific effect that becomes more pronounced in the later phases of the postintervention period.

Hence, modulation effects between the low-frequency and repeated low-frequency protocols were compared by normalizing P1N1 values to individual baselines and analyzing the normalized values via two-way ANOVA (Fig. 2H). The results revealed a significant main effect between protocols (*F*(1, 449) = 33.93, *p* < 0.0001). The effect of the time factor was also found to be significant (*F*(5, 449) = 3.013, *p* = 0.0109). Post hoc analysis revealed significant differences at 2 min (mean difference = −43.40, *p* < 0.0001) and 22 min postmodulation (mean difference = −34.10, *p* = 0.0054). No significant differences were observed at the other time points. These findings indicate that the repeated low-frequency protocol consistently produced higher P1N1 values than did the low-frequency protocol, with significant differences in the effects of the two protocols observed at 2 and 22 min postmodulation. The significant main effects of protocol and time reflect distinct protocol-specific and temporal effects. These results highlight the sustained efficacy of the repeated low-frequency protocol.

### High-frequency modulation induces strong but transient changes

The high-frequency modulation protocol induced a strong, immediate increase in P1N1 at 2 min postmodulation, as shown by the grand average VEP traces in Fig. 3C. This effect was confirmed via repeated measures one-way ANOVA, which revealed a significant difference across time points (*F*(4.928, 138.0) = 13.86, *p* < 0.0001). Post hoc tests revealed a significant increase in P1N1 compared with that at baseline at 2 min postmodulation (mean difference = −5.712, *p* < 0.0001) and a smaller, but still significant, increase at 12 min postmodulation (mean difference = −2.254, *p* = 0.0215). No significant differences were observed at the later time points. These results suggest that the high-frequency protocol induces a strong but brief increase in P1N1 that decreases quickly over time.

### Theta-pulse modulation induces moderate but sustained changes

The theta-pulse modulation protocol was shown to induce sustained increases in P1N1 compared with that at baseline, as shown by the grand average VEP traces in Fig. 3D. A repeated-measures ANOVA revealed a significant effect of the protocol on P1N1 across time points (F(3.875, 116.3) = 4.379, p = 0.0027). Post hoc tests revealed significant increases in P1N1 at several time points postintervention. A notable increase in P1N1 was observed immediately at 2 min postmodulation (mean difference = −2.941, p = 0.0002), with further significant increases observed at 8 min (mean difference = −1.806, p = 0.0368), 18 min (mean difference = −2.334, p = 0.0058), 22 min (mean difference = −1.952, p = 0.0224), and 28 min postmodulation (mean difference = −1.719, p = 0.0374). No significant difference was observed at 12 min postmodulation (mean difference = −1.444, p = 0.0867). These results suggest that the theta-pulse modulation protocol induces prolonged increases in P1N1, with the most pronounced effects occurring immediately after the intervention and the effects persisting over time.

### High-frequency vs. theta-pulse modulation

To assess differences in the modulation effects between the high-frequency and theta-pulse protocols, the baseline P1N1 value was subtracted from the P1N1 values obtained in the early and late postmodulation periods for each individual (Fig. 3G). An unpaired *t* test revealed a statistically significant difference in the P1N1 value obtained in the early modulation period between the protocols (*t*(57) = 2.734, *p* = 0.0083). The mean P1N1 change was significantly greater in the pulse condition (mean = 5.727) than in the theta condition (mean = 2.938), with a mean difference of −2.788 ± 1.020. In contrast, a comparison of the average change in the P1N1 value in the late modulation phase revealed no significant difference between the pulse and theta conditions (*t*(57) = 0.6322, *p* = 0.5298). The mean P1N1 values obtained under the pulse and theta conditions were 1.277 and 1.806, respectively, with a mean difference of 0.5288 ± 0.8365.

Data from the high-frequency and theta-pulse protocols were normalized to individual baselines and analyzed via two-way ANOVA (Fig. 3H), which was used to evaluate interaction effects between the time points and protocols. The analysis revealed a statistically significant interaction effect between the time points and protocols (*F*(5, 322) = 3.123, *p* = 0.0091), as well as a significant main effect of time (*F*(5, 322) = 4.233, *p* = 0.0010). However, no significant differences between the protocols were found (*F*(1, 322) = 0.5111, *p* = 0.4752). Exploratory post hoc tests indicated a significant difference between the protocols at 2 min postmodulation (*p* = 0.0129); however, no significant differences were observed at later time points. These findings indicate that the interaction effects between the protocols and time points are driven primarily by the significant differences observed at 2 min postmodulation, with better effects achieved with the high-frequency modulation protocol. The significance of the row factor underscores the time-dependent changes observed across both groups.

### Temporal stability and consistency

**Fig. 4:**
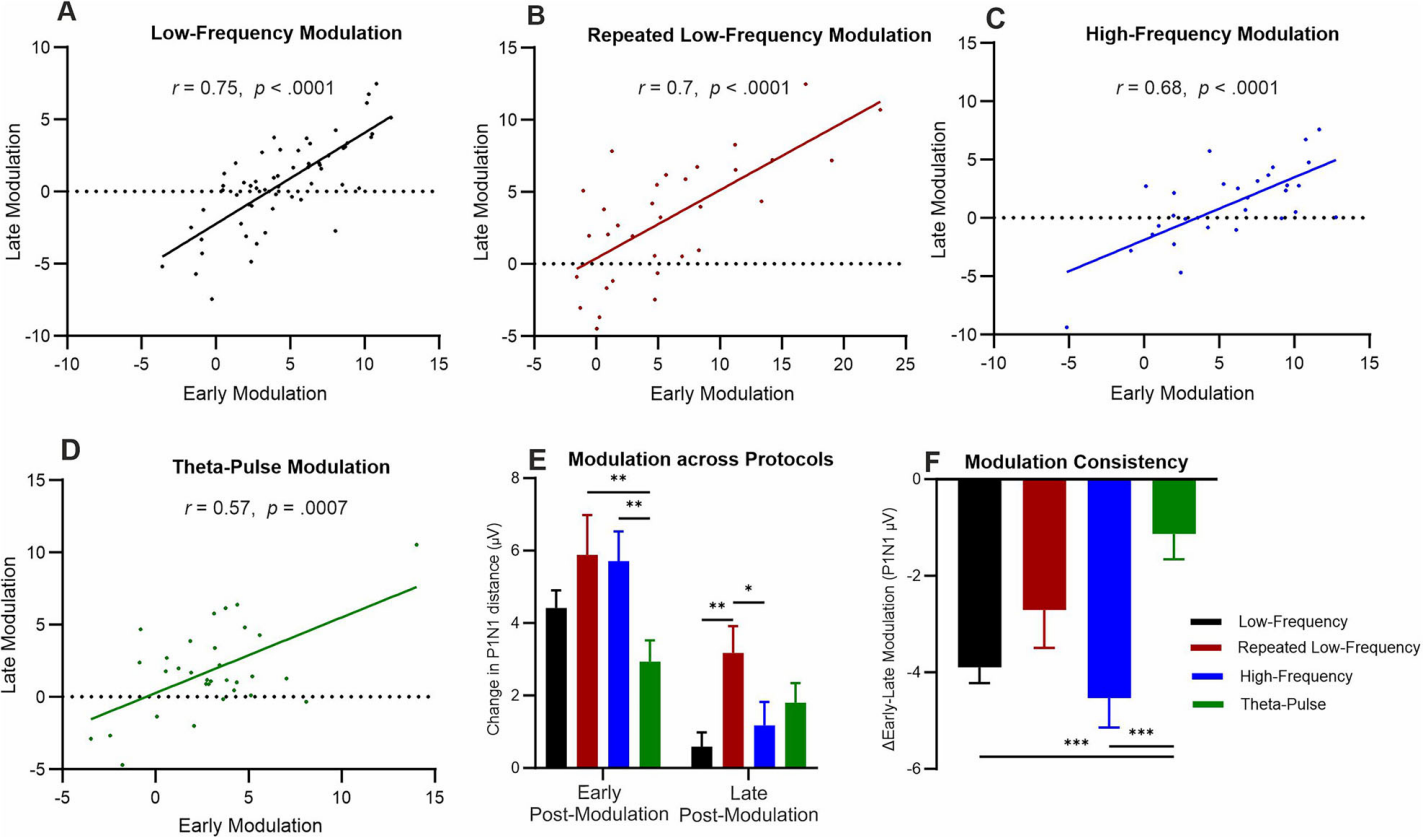
Temporal stability and modulation consistency of the P1N1 amplitude across different stimulation protocols. **A**–**D** Scatter plots showing the correlation between the P1N1 modulation in the early (2 min) and late (Ø 22 and 28 min) postmodulation periods for each protocol: **A** Low-frequency modulation (*n* = 57), **B** repeated low-frequency modulation (*n* = 34), **C** high-frequency modulation (*n* = 29), and **D** theta-pulse modulation (*n* = 32). Pearson correlation coefficients (*r*) and significance values are indicated for each protocol. **E** Bar graph depicting the mean change in the P1N1 amplitude (in µV) for early and late postmodulation time points across protocols. **F** Modulation consistency (ΔEarly-Late Modulation) across different stimulation conditions. Post hoc comparisons revealed significant differences between the low-frequency vs. theta-pulse (*p* = 0.0008) and high-frequency vs. theta-pulse (*p* = 0.0004) conditions. The data are presented as the means ± SEMs. *p < 0.05, **p < 0.01, ***p < 0.001.

To assess the stability of early and late P1N1 modulation over time, Pearson correlation analyses were performed with the data for each protocol. Significant positive correlations were found for all protocols, highlighting that the modulation effects in the immediate postintervention phase and later time points were consistent. For the low-frequency protocol, the correlation between the P1N1 values obtained in the early (2 min postmodulation) and late (22 and 28 min postmodulation) postmodulation periods was strong (*r* = 0.75, *p* < 0.0001; Fig. 4A). Similar results were found for the repeated low-frequency protocol (*r* = 0.7, *p* < 0.0001; Fig. 4B), the high-frequency protocol (*r* = 0.68, *p* < 0.0001; Fig. 4C) and the theta-pulse protocol, but the correlations were slightly weaker (*r* = 0.57, *p* = 0.0007; Fig. 4D).

Figure 4 shows a summary of the average P1N1 modulation amplitude changes across protocols in both the early and late postmodulation phases. Compared with the effects observed in the late modulation period, the effects in the early modulation period were significantly greater for all the protocols. Notably, the smallest modulation changes across both time points were found for the theta-pulse protocol. Two-way ANOVA revealed significant main effects of time (*F*(1297) = 42.07, *p* < 0.0001) and protocol (*F*(3297) = 4.58, *p* = 0.0038), indicating that VEP changes varied across time points and stimulation conditions. Post hoc tests revealed significant differences in the P1N1 changes in the early modulation among protocols (high-frequency vs. theta-pulse, *p* = 0.0064; theta-pulse vs. repeated low-frequency, *p* = 0.0031), highlighting temporal differences in early plasticity expression. In the late modulation phase, the P1N1 value in the repeated low-frequency stimulation condition was found to be significantly greater than those in both the high-frequency (*p* = 0.049) and low-frequency stimulation conditions (*p* = 0.0029).

Figure 4F shows a summary of the modulation consistency (ΔEarly-Late Modulation) across protocols. One-way ANOVA revealed a significant effect of protocol on modulation consistency (*F*(3207) = 7.207, *p* = 0.0002). Post hoc comparisons revealed that the theta-pulse stimulation protocol was associated with significantly greater consistency (closer to zero) than were the low-frequency (*p* = 0.0008) and high-frequency (*p* = 0.0004) stimulation protocols. These results suggest that the theta-pulse stimulation protocol is associated with more stable modulation effects over time, whereas more varied effects are observed with the other protocols in the early and late modulation stages. These analyses suggest that, at the individual level, the extent of late plastic effects can be detected in the early postmodulation period using the repeated low-frequency and theta-pulse stimulation protocols, thus reducing the need for long experimental protocols.

## Discussion

In this study, we systematically examined the impact of four modulation protocols—low-frequency, repeated low-frequency, high-frequency, and theta-pulse stimulation—on changes in VEPs, highlighting the critical influence of the stimulation frequency and pattern on plasticity outcomes. Our four key findings can be summarized as follows: (1) Low-frequency stimulation induced short-term increases in P1N1 amplitudes that rapidly decreased to baseline levels, whereas repeated low-frequency stimulation led to more sustained changes in excitability. (2) High-frequency stimulation produced sharp but transient excitability increases, whereas theta-pulse stimulation generated consistent and prolonged effects. (3) Compared with the single low-frequency and high-frequency stimulation protocols, the repeated low-frequency and theta-pulse protocols are more effective in maintaining long-term plasticity. (4) As early and late stage postmodulation effects are clearly correlated, prolonged assessments may not be necessary.

### Low-frequency and repeated low-frequency modulation

Low-frequency modulation produced a transient increase in P1N1 values, with the P1N1 values returning to baseline levels within 12 min. This corresponds to short-term rather than long-term changes in neuronal architecture [4]; thus, single low-frequency modulation is a suboptimal protocol for inducing long-term plasticity changes. In contrast, the repeated low-frequency modulation protocol elicited more sustained changes in P1N1, which persisted until the late postintervention phases (22 min postmodulation). It is plausible that the extended 30 min stimulation phase facilitated the synchronization of neuronal oscillations in the primary visual cortex, resulting in more durable plasticity changes. Therefore, repeated low-frequency stimulation phases should be incorporated in protocols developed to induce sustained changes, especially for clinical applications aimed at providing rehabilitative or therapeutic outcomes.

### High-frequency modulation and theta-pulse modulation

High-frequency modulation produced sharp increases in P1N1 amplitudes immediately following stimulation; however, these effects were transient, with excitability returning to baseline shortly after the intervention. This outcome aligns with previous findings, suggesting that while high-frequency stimulation can effectively induce STP, the sustained presynaptic calcium dynamics and downstream signaling cascades required for LTP may be lacking with this protocol [5, 23]. Consequently, high-frequency stimulation protocols may be more suitable for applications requiring rapid but temporary neural activation rather than sustained plasticity changes.

In contrast, the theta-pulse modulation protocol was associated with consistent and prolonged changes in VEP amplitudes, with changes persisting until the late postintervention phases. This finding highlights the potential of theta-pulse stimulation to reliably induce long-term plasticity changes. In theta-pulse protocols, the intrinsic oscillatory rhythms of the brain, particularly those in the theta-frequency range, are likely leveraged; importantly, these oscillatory rhythms are known to facilitate synaptic synchronization and increase the induction of LTP in hippocampal and cortical circuits [21, 45]. By aligning the stimulation protocol with these natural neural oscillations, theta-pulse protocols may promote more stable and enduring synaptic modifications [10]. In repetitive transcranial magnetic stimulation (rTMS), intermittent theta-burst protocols have largely replaced the previously used continuous protocols [46]. Thus, rhythmic stimulation patterns that resonate with the brain’s endogenous activity may be critical inducing visual LTP. Additionally, the prolonged effects observed with theta-pulse modulation underscore its potential utility in clinical settings, where durable plasticity changes are essential for ensuring the efficacy of therapeutic interventions. Future studies should explore how varying the parameters of theta-pulse stimulation, such as the burst duration or interpulse intervals, might further optimize its effectiveness.

## Limitations

This study has several limitations that should be considered. First, we used a small number of electrodes, which provides only limited information on the spatial changes in the VEPs. Second, our sample consisted of only young, healthy and primarily female adults, which could lead to sex-related biases, limiting the generalizability of the findings to older populations and other demographic groups. We also did not control for menstrual cycle phase, which may introduce additional variability, although prior work found no reliable changes in sensory LTP across the cycle [47]. Third, it remains unclear whether pattern-reversal and onset–offset paradigms engage plasticity mechanisms differently at matched frequencies. Existing literature supports that each contrast change elicits a distinct cortical response at the full reversal frequency, and our analysis of black-to-white (trigger type 1) and white-to-black (trigger type 2) transitions confirmed identical responses. Nonetheless, some uncertainty remains without direct comparisons to onset–offset paradigms. Fourth, we cannot fully exclude that the brief 2 rps test sequences have partially influenced the plasticity effects; however, the consistent pattern and direction of potentiation observed across all protocols argue against a substantial impact of the testing stimulation.

Finally, study remains primarily descriptive, though it parallels animal research showing that theta-burst stimulation induces plasticity in both the hippocampus and the visual system [48, 49]. The visual cortex, however, exhibits distinct oscillatory properties, with the alpha frequency band likely playing an important role; it is not yet clear whether this reflects true synaptic plasticity, frequency-specific entrainment, or contextual modulation [10]. Moreover, our recordings were confined to primary visual areas and thus may not reflect changes in higher-order associative regions. The methodological limitations of surface VEPs preclude disentangling synaptic plasticity from entrainment effects or shifts in excitation-inhibition balance, warranting further investigation.

## Conclusion

However, our findings demonstrate clear differences in how the modulation length, frequency and pattern influence short-term and long-term changes in LTP-like plasticity in the visual cortex. Distinct dynamics were observed with the high-frequency and theta-pulse protocols, with high-frequency modulation inducing sharp but transient changes and theta-pulse modulation generating more consistent effects over time. These results provide new insights into the temporal and frequency-specific dynamics of cortical plasticity and address the variability observed in previous studies.

### Implications for the use of vep-related plasticity as a biomarker in psychiatry

**Fig. 5:**
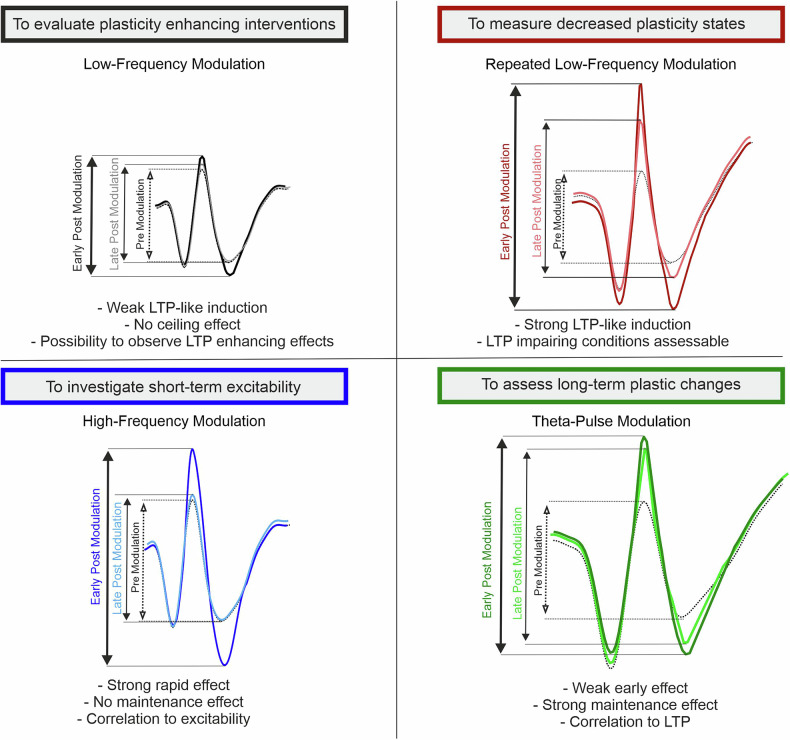
Stimulation protocols and corresponding VEP changes. All panels show schematic VEP traces with the premodulation trace indicated by the dashed line, along with the traces in the early and late postmodulation phases. Upper left: Low-frequency stimulation protocol. Upper right: Repeated low-frequency stimulation protocol. Lower left: High-frequency stimulation protocol. Lower right: Theta pulse stimulation protocol. These data demonstrate how different stimulation protocols can be used to assess various aspects of neuroplasticity and excitability.

Previous studies have revealed robust group effects on VEP-related plasticity among individuals with disorders involving dysregulated neuroplasticity, particularly major depression disorder and schizophrenia. Moreover, psychopharmacological drugs have been found to modulate VEP-related plasticity at the group level. To use VEP-related plasticity as a biomarker at the individual level, several aspects must be considered [50]:

- For diagnostic purposes, the validity, reliability, and predictive power of VEP-related plasticity, including its adequate sensitivity and specificity in differentiating between healthy individuals and those with specific disorders, must be determined through larger-scale studies.
- The assessments of the VEP-related plasticity protocols should be feasible and viable outside research laboratory settings. This includes the validation of shorter protocols, minimizing the time needed for the modulation protocol and the postmodulation assessments. Moreover, advanced EEG techniques and user-friendly mobile health tools are crucial for the application of these protocols in real-world settings [51].
- We hypothesize that early postmodulation changes reflect changes in excitability, whereas later changes indicate LTP-like plasticity effects. Additional trials using specific pharmacological interventions are needed to determine whether the two putative components of the SRP response can be modulated independently.
- The choice of a suitable stimulation protocol depends on the intended use of the putative VEP biomarker (Fig. 5). As a diagnostic tool for disorders in which impaired plasticity is assumed, a stimulation protocol that produces maximally high effects in healthy controls should be used. In contrast, to assess the effects of drugs and interventions aimed at increasing plasticity, a submaximal protocol, such as the low-frequency protocol, would be advantageous. To evaluate interventions aimed at long-lasting plastic effects, the theta-pulse protocol should be used; furthermore, to evaluate post-tetanic changes in excitability, the high-frequency stimulation protocol should be applied (Fig. 5).

Our study provides the first insights into strategies to optimize VEP-related plasticity as a future biomarker for the diagnosis of plasticity-dependent disorders and for the development of drugs and prediction of the responses of plasticity-modulating agents, supporting its potential clinical translation into individualized treatment planning.

## Supplementary information

Supplemental material

## Data Availability

The data analyzed in this study are not publicly available for reasons of confidentiality but can be requested from the corresponding author upon reasonable request.

## References

[1] Appelbaum LG, Shenasa MA, Stolz L, Daskalakis Z. Synaptic plasticity and mental health: methods, challenges and opportunities. Neuropsychopharmacol. 2023;48:113–20. 10.1038/s41386-022-01370-w PMC9700665 35810199

[2] von Bernhardi R, Bernhardi LE, Eugenín J. What is neural plasticity?. Adv Exp Med Biol. 2017;1015:1–15. 29080018 10.1007/978-3-319-62817-2_1

[3] Citri A, Malenka RC. Synaptic plasticity: multiple forms, functions, and mechanisms. Neuropsychopharmacol. 2008;33:18–41. 10.1038/sj.npp.1301559 17728696

[4] Schulz PE, Fitzgibbons JC. Differing mechanisms of expression for short- and long-term potentiation. J Neurophysiol. 1997;78:321–34. 9242283 10.1152/jn.1997.78.1.321

[5] Bliss TV, Collingridge GL. A synaptic model of memory: long-term potentiation in the hippocampus. Nature. 1993;361:31–39. 8421494 10.1038/361031a0

[6] Herzberg MP, Nielsen AN, Luby J, Sylvester CM. Measuring neuroplasticity in human development: the potential to inform the type and timing of mental health interventions. Neuropsychopharmacol. 2024;50:124–36. 10.1038/s41386-024-01947-7 PMC11525577 39103496

[7] Sanders PJ, Thompson B, Corballis PM, Maslin M, Searchfield GD. A review of plasticity induced by auditory and visual tetanic stimulation in humans. Eur J Neurosci. 2018;48:2084–97. 30025183 10.1111/ejn.14080

[8] Sumner RL, Spriggs MJ, Muthukumaraswamy SD, Kirk IJ. The role of Hebbian learning in human perception: a methodological and theoretical review of the human Visual Long-Term Potentiation paradigm. Neurosci Biobehav Rev. 2020;115:220–37. 32562886 10.1016/j.neubiorev.2020.03.013

[9] Kirk IJ, Spriggs MJ, Sumner RL. Human EEG and the mechanisms of memory: investigating long-term potentiation (LTP) in sensory-evoked potentials. J R Soc N. Zealand. 2021;51:24–40.

[10] Kirk IJ, McNair NA, Hamm JP, Clapp WC, Mathalon DH, Cavus I, et al. Long-term potentiation (LTP) of human sensory-evoked potentials. WIREs Cogn Sci. 2010;1:766–73. 10.1002/wcs.62 26271660

[11] Cooke SF, Bear MF. Visual experience induces long-term potentiation in the primary visual cortex. J Neurosci. 2010;30:16304–13. 21123576 10.1523/JNEUROSCI.4333-10.2010 PMC3078625

[12] Frenkel MY, Sawtell NB, Diogo ACM, Yoon B, Neve RL, Bear MF. Instructive effect of visual experience in mouse visual cortex. Neuron. 2006;51:339–49. 16880128 10.1016/j.neuron.2006.06.026

[13] Teyler TJ, Hamm JP, Clapp WC, Johnson BW, Corballis MC, Kirk IJ. Long-term potentiation of human visual evoked responses. Eur J Neurosci. 2005;21:2045–50. 15869500 10.1111/j.1460-9568.2005.04007.x PMC1226326

[14] Normann C, Schmitz D, Fürmaier A, Döing C, Bach M. Long-term plasticity of visually evoked potentials in humans is altered in major depression. Biol Psychiatry. 2007;62:373–80. 17240361 10.1016/j.biopsych.2006.10.006

[15] Forsyth JK, Bachman P, Mathalon DH, Roach BJ, Asarnow RF. Augmenting NMDA receptor signaling boosts experience-dependent neuroplasticity in the adult human brain. Proc Natl Acad Sci USA. 2015;112:15331–6. 26621715 10.1073/pnas.1509262112 PMC4687562

[16] McNair NA, Clapp WC, Hamm JP, Teyler TJ, Corballis MC, Kirk IJ. Spatial frequency-specific potentiation of human visual-evoked potentials. Neuroreport. 2006;17:739–41. 16641679 10.1097/01.wnr.0000215775.53732.9f

[17] Ross RM, McNair NA, Fairhall SL, Clapp WC, Hamm JP, Teyler TJ, et al. Induction of orientation-specific LTP-like changes in human visual evoked potentials by rapid sensory stimulation. Brain Res Bull. 2008;76:97–101. 18395617 10.1016/j.brainresbull.2008.01.021

[18] Cooke SF, Bear MF. Stimulus-selective response plasticity in the visual cortex: an assay for the assessment of pathophysiology and treatment of cognitive impairment associated with psychiatric disorders. Biol Psychiatry. 2012;71:487–95. 22019003 10.1016/j.biopsych.2011.09.006

[19] Tsanov M, Manahan-Vaughan D. Visual cortex plasticity evokes excitatory alterations in the hippocampus. Front Integr Neurosci. 2009;3:32. 19956399 10.3389/neuro.07.032.2009 PMC2786298

[20] Tsanov M, Manahan-Vaughan D. Synaptic plasticity from visual cortex to hippocampus: systems integration in spatial information processing. Neuroscientist. 2008;14:584–97. 18612086 10.1177/1073858408315655

[21] Spriggs MJ, Thompson CS, Moreau D, McNair NA, Wu CC, Lamb YN, et al. Human sensory LTP predicts memory performance and is modulated by the BDNF Val66Met polymorphism. Front Hum Neurosci. 2019;13:22. 30828292 10.3389/fnhum.2019.00022 PMC6384276

[22] Dias JW, McClaskey CM, Rumschlag JA, Harris KC. Sensory tetanization to induce long-term-potentiation-like plasticity: a review and reassessment of the approach. Eur J Neurosci. 2022;56:6115. 36227258 10.1111/ejn.15847 PMC9772088

[23] Zucker RS, Regehr WG. Short-term synaptic plasticity. Annu Rev Physiol. 2002;64:355–405. 11826273 10.1146/annurev.physiol.64.092501.114547

[24] Magleby KL, Zengel JE. A quantitative description of stimulation-induced changes in transmitter release at the frog neuromuscular junction. J Gen Physiol. 1982;80:613–38. 6128373 10.1085/jgp.80.4.613 PMC2228704

[25] Dudek SM, Bear MF. Homosynaptic long-term depression in area CA1 of hippocampus and effects of N-methyl-D-aspartate receptor blockade. Proc Natl Acad Sci USA. 1992;89:4363–7. 1350090 10.1073/pnas.89.10.4363 PMC49082

[26] Mulkey RM, Malenka RC. Mechanisms underlying induction of homosynaptic long-term depression in area CA1 of the hippocampus. Neuron. 1992;9:967–75. 1419003 10.1016/0896-6273(92)90248-c

[27] Larson J, Munkácsy E. Theta-burst LTP. Brain Res. 2015;1621:38–50. 25452022 10.1016/j.brainres.2014.10.034 PMC4411212

[28] Rygvold TW, Hatlestad-Hall C, Elvsåshagen T, Moberget T, Andersson S. Long-term potentiation-like visual synaptic plasticity is negatively associated with self-reported symptoms of depression and stress in healthy adults. Front Hum Neurosci. 2022;16:867675. 35601905 10.3389/fnhum.2022.867675 PMC9119023

[29] Valstad M, Roelfs D, Slapø NB, Timpe CMF, Rai A, Matziorinis AM, et al. Evidence for reduced long-term potentiation-like visual cortical plasticity in schizophrenia and bipolar disorder. Schizophr Bull. 2021;47:1751–60. 33963856 10.1093/schbul/sbab049 PMC8530383

[30] Zak N, Moberget T, Bøen E, Boye B, Waage TR, Dietrichs E, et al. Longitudinal and cross-sectional investigations of long-term potentiation-like cortical plasticity in bipolar disorder type II and healthy individuals. Transl Psychiatry. 2018;8:1–12. 29795193 10.1038/s41398-018-0151-5 PMC5966393

[31] Jacob MS, Roach BJ, Hamilton HK, Carrión RE, Belger A, Duncan E, et al. Visual cortical plasticity and the risk for psychosis: an interim analysis of the North American prodrome longitudinal study. Schizophr Res. 2021;230:26–37. 33667856 10.1016/j.schres.2021.01.028 PMC8328744

[32] Wynn JK, Roach BJ, McCleery A, Marder SR, Mathalon DH, Green MF. Evaluating visual neuroplasticity with EEG in schizophrenia outpatients. Schizophr Res. 2019;212:40–46. 31434625 10.1016/j.schres.2019.08.015 PMC6791734

[33] Cavuş I, Reinhart RMG, Roach BJ, Gueorguieva R, Teyler TJ, Clapp WC, et al. Impaired visual cortical plasticity in schizophrenia. Biol Psychiatry. 2012;71:512–20. 22364738 10.1016/j.biopsych.2012.01.013 PMC3292767

[34] Spriggs MJ, Cadwallader CJ, Hamm JP, Tippett LJ, Kirk IJ. Age-related alterations in human neocortical plasticity. Brain Res Bull. 2017;130:53–59. 28043855 10.1016/j.brainresbull.2016.12.015

[35] Sumner RL, McMillan R, Spriggs MJ, Campbell D, Malpas G, Maxwell E, et al. Ketamine enhances visual sensory evoked potential long-term potentiation in patients with major depressive disorder. Biol Psychiatry Cogn Neurosci Neuroimaging. 2020;5:45–55. 31495712 10.1016/j.bpsc.2019.07.002

[36] Frase L, Mertens L, Krahl A, Bhatia K, Feige B, Heinrich SP, et al. Transcranial direct current stimulation induces long-term potentiation-like plasticity in the human visual cortex. Transl Psychiatry. 2021;11:17. 33414402 10.1038/s41398-020-01134-4 PMC7791098

[37] Kuhn M, Wolf E, Maier JG, Mainberger F, Feige B, Schmid H, et al. Sleep recalibrates homeostatic and associative synaptic plasticity in the human cortex. Nat Commun. 2016;7:12455. 27551934 10.1038/ncomms12455 PMC4996971

[38] Wolf E, Kuhn M, Normann C, Mainberger F, Maier JG, Maywald S, et al. Synaptic plasticity model of therapeutic sleep deprivation in major depression. Sleep Med Rev. 2016;30:53–62. 26803484 10.1016/j.smrv.2015.11.003

[39] Krause F, Lindemann O. Expyriment: a python library for cognitive and neuroscientific experiments. Behav Res Methods. 2014;46:416–28. 24142834 10.3758/s13428-013-0390-6

[40] Seitz A, Watanabe T. A unified model for perceptual learning. Trends Cogn Sci. 2005;9:329–34. 15955722 10.1016/j.tics.2005.05.010

[41] Elvsåshagen T, Moberget T, Bøen E, Boye B, Englin NOA, Pedersen PØ, et al. Evidence for impaired neocortical synaptic plasticity in bipolar II disorder. Biol Psychiatry. 2012;71:68–74. 22036034 10.1016/j.biopsych.2011.09.026

[42] Valstad M, Moberget T, Roelfs D, Slapø NB, Timpe CMF, Beck D, et al. Experience-dependent modulation of the visual evoked potential: testing effect sizes, retention over time, and associations with age in 415 healthy individuals. Neuroimage. 2020;223:117302. 32828930 10.1016/j.neuroimage.2020.117302

[43] Hohberger B, Kremers J, Horn FK. Steady-state visually evoked potentials elicited by multifrequency pattern-reversal stimulation. Transl Vis Sci Technol. 2019;8:24. 30834172 10.1167/tvst.8.1.24 PMC6396688

[44] Norcia AM, Appelbaum LG, Ales JM, Cottereau BR, Rossion B. The steady-state visual evoked potential in vision research: a review. J Vis. 2015;15:4. 26024451 10.1167/15.6.4 PMC4581566

[45] Clapp WC, Hamm JP, Kirk IJ, Teyler TJ. Translating LTP from animals to humans: a novel method for non-invasive assessment of cortical plasticity. Biol Psychiatry. 2012;71:496–502. 21974785 10.1016/j.biopsych.2011.08.021 PMC3253317

[46] Kishi T, Ikuta T, Sakuma K, Hatano M, Matsuda Y, Wilkening J, et al. Theta burst stimulation for depression: a systematic review and network and pairwise meta-analysis. Mol Psychiatry. 2024;29:3893–9. 38844532 10.1038/s41380-024-02630-5 PMC11609094

[47] Sumner RL, Spriggs MJ, McMillan RL, Sundram F, Kirk IJ, Muthukumaraswamy SD. Neural plasticity is modified over the human menstrual cycle: Combined insight from sensory evoked potential LTP and repetition suppression. Neurobiol Learn Mem. 2018;155:422–34. 30172951 10.1016/j.nlm.2018.08.016

[48] Kirkwood A, Rioult MC, Bear MF. Experience-dependent modification of synaptic plasticity in visual cortex. Nature. 1996;381:526–8. 8632826 10.1038/381526a0

[49] Heynen AJ, Bear MF. Long-term potentiation of thalamocortical transmission in the adult visual cortex in vivo. J Neurosci. 2001;21:9801–13. 11739588 10.1523/JNEUROSCI.21-24-09801.2001 PMC6763060

[50] Abi-Dargham A, Moeller SJ, Ali F, DeLorenzo C, Domschke K, Horga G, et al. Candidate biomarkers in psychiatric disorders: state of the field. World Psychiatry. 2023;22:236–62. 37159365 10.1002/wps.21078 PMC10168176

[51] Barbey FM, Farina FR, Buick AR, Danyeli L, Dyer JF, Islam MN, et al. Neuroscience from the comfort of your home: repeated, self-administered wireless dry EEG measures brain function with high fidelity. Front Digit Health. 2022;4:944753. 35966140 10.3389/fdgth.2022.944753 PMC9372279

