# Supplementary material for: Development of VEP-based biomarkers to assess plasticity states
Source: Transl Psychiatry. 2025 Oct 20;15:426. doi: 10.1038/s41398-025-03676-x (PMC12537939; doi:10.1038/s41398-025-03676-x)
Supplement: Supplementary file 1 — Supplemental material [file 41398_2025_3676_MOESM1_ESM.pdf]

Supplementary Material

Development of VEP-Based Biomarkers to Assess Plasticity States

Figures

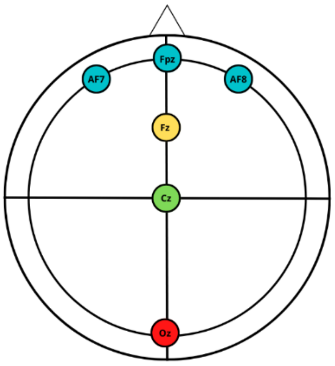

Figure S1. Schematic illustration of the electrode set-up.

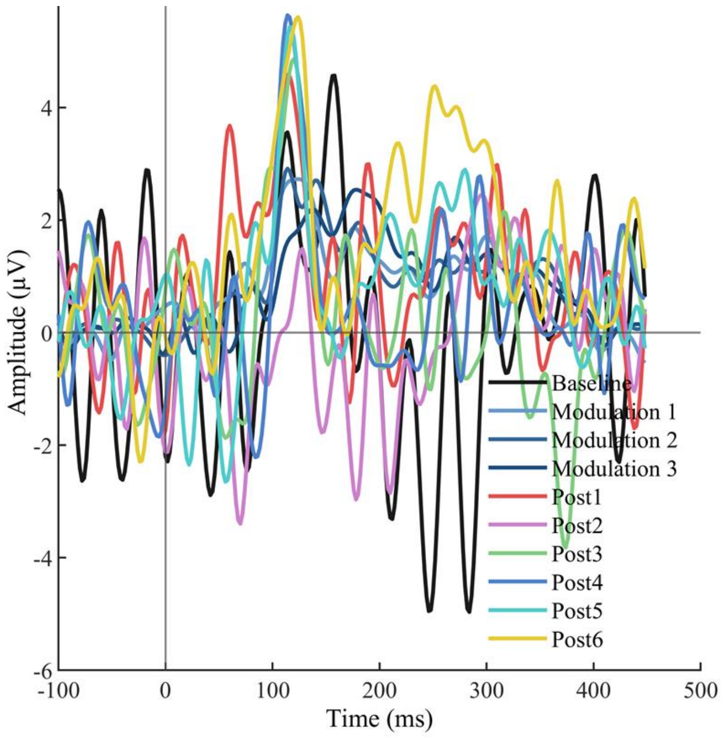

Figure S2. Exemplary figure of a manually rejected measurement due to an erratic, non-VEP-consistent pattern.

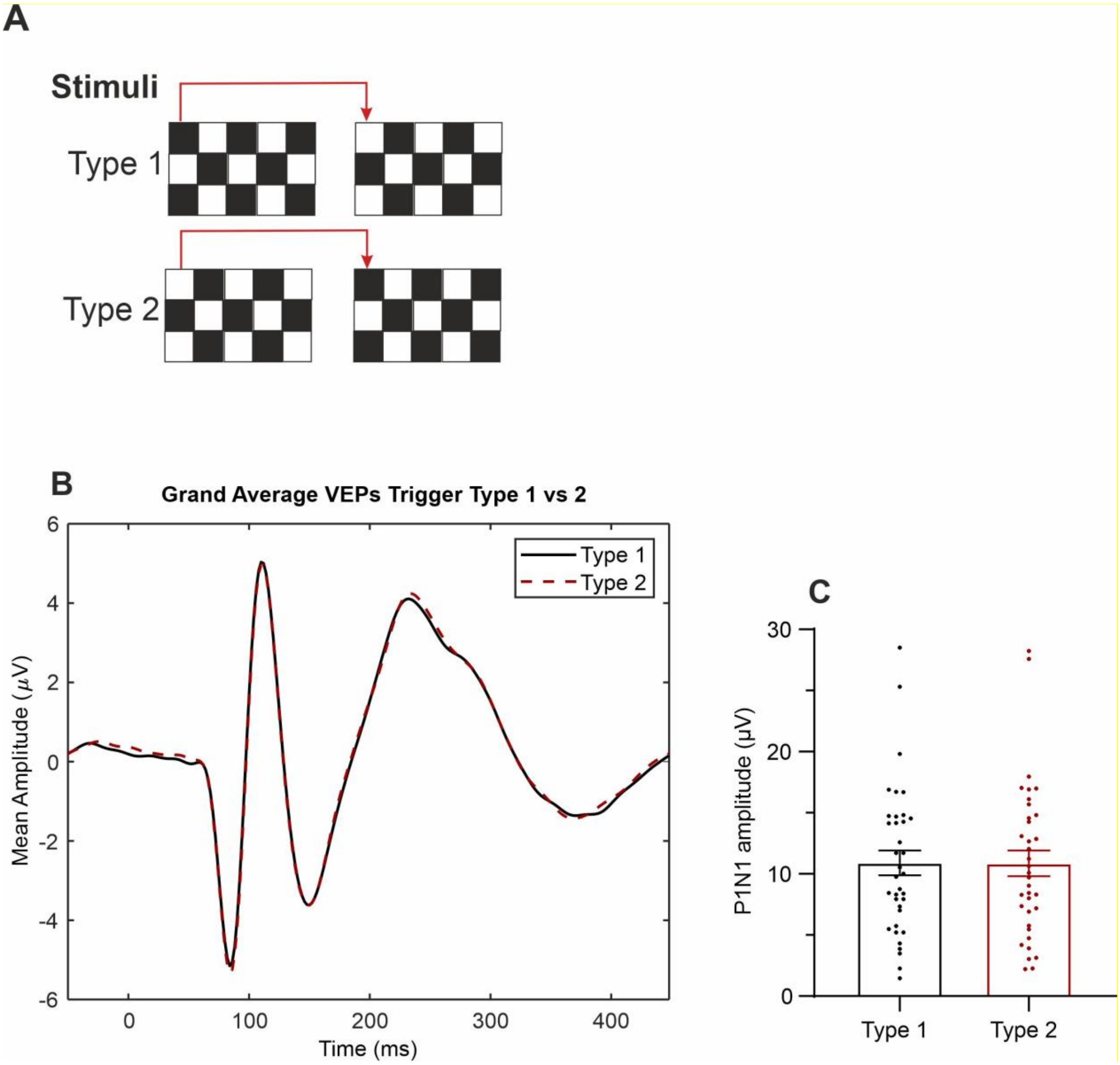

Figure S3. Comparison of trigger types 1 and 2 (checkerboard reversal).
A Schematic illustration of the difference between trigger type 1 and 2 by index fields. **B** Epochs were collected from a 10-minute 2 rps modulation phase (*n* = 33 participants), resulting in ~1200 stimuli per participant (600 per trigger type). Epochs were averaged per individual and trigger type; shown are the grand average group waveforms (mean across participants). **C** P1N1 amplitudes were extracted from each participant's average waveform (based on 600 epochs per trigger type). Shown are the group means and individual participant data points (*n* = 33) for trigger types 1 and 2. Equivalence testing confirmed no meaningful differences between conditions (TOST, SESOI ±0.5 μV, *p*-lower = .0005*, p*-upper = .0033).

Tables

Demographic Characteristics

Table S1. Baseline demographic comparison of study groups.

		Study group 1[a]	Study group 2[b]	
		***N* = 68**	***N* = 36**	***p***
Age, years, mean ± SD[c]		28.84 ± 8.99	27.08 ± 4.01	.2691
Gender, *n* (%)[d]	female	42 (61.76%)	23 (63.89%)	>.9999
	male	26 (38.24%)	13 (36.11%)	

[a] Study group 1 underwent low-frequency protocol.
[b] Study group 2 participated in a within-subject design with repeated-low-frequency, high-frequency, and theta-pulse protocols.
[c] Unpaired t test
[d] Fisher's exact test

Component results

Table S2. Mean (M) and Standard Deviation (SD) of C1 Values Across Protocols and Time Points with Within-Protocol Modulation Results

Protocol		Baseline	Post 1	Post 2	Post 3	Post 4	Post 5	Post 6	F-value	p-value
Low-Frequency (*n* = 57)	M	-6.749	-5.401	-6.092	-6.175	-6.53	-6.428	-6.518	F (4.796. 256.6) = 2.636	.0259
	SD	5.508	4.676	5.224	4.818	5.587	4.675	5.321		
Repeated low-frequency (*n* = 33)	M	-8.018	-4.5****	-5.412**	-6.317	-6.447	-6.494	-6.628	F (4.235. 132.0) = 6.490	<.0001
	SD	6.065	4.766	5.807	5.669	5.743	4.718	5.204		
High-frequency (*n* = 29)	M	-8.21	-4.492***	-4.565**	-6.19*	-6.431	-6.168*	-5.912*	F (4.240. 117.3) = 4.262	.0024
	SD	6.035	4.773	3.656	5.553	5.253	5.143	6.088		
Theta-Pulse (*n* = 32)	M	-7.694	-6.175	-6.019	-6.481	-5.918	-6.19	-5.677*	F (4.225. 131.0) = 2.942	.0208
	SD	5.663	5.1	4.212	4.752	3.708	4.764	4.834		

Note. Row effects were analyzed using one-way ANOVA with repeated measures to compare each post time point against the baseline. A restricted maximum likelihood (REML) model was fitted when data were missing. Stars (*, **, ***, ****) indicate significant post-hoc comparisons using Dunnett's multiple comparisons test.

Table S3. Mean (M) and Standard Deviation (SD) of P1 Values Across Protocols and Time Points with Within-Protocol Modulation Results

Protocol		Baseline	Post 1	Post 2	Post 3	Post 4	Post 5	Post 6	F-value	p-value
Low-Frequency (*n* = 57)	M	5.453	9.166****	7.312**	7.334***	6.417	6.308	6.45	F (4.431. 238.5) = 14.17	<.0001
	SD	5.804	5.18	5.164	5.507	5.222	5.084	5.61		
Repeated low-frequency (*n* = 33)	M	3.202	8.886****	7.655****	6.059**	5.528**	5.673**	5.267*	F (5.004. 156.8) = 15.15	<.0001
	SD	5.338	6.645	6.136	6.461	6.154	5.311	6.579		
High-frequency (*n* = 29)	M	3.004	7.593****	5.563**	4.532	4.308	4.699	4.859	F (4.623. 129.4) = 7.388	<.0001
	SD	4.807	5.896	5.343	5.144	5.663	5.916	6.725		
Theta-Pulse (*n* = 32)	M	3.191	5.514*	5.133*	4.032	5.053*	4.351	4.573	F (5.190. 160.9) = 3.200	.0080
	SD	5.931	6.013	6.156	6.774	5.723	5.775	5.79		

Note. Row effects were analyzed using one-way ANOVA with repeated measures to compare each post time point against the baseline. A restricted maximum likelihood (REML) model was fitted when data were missing. Stars (*, **, ***, ****) indicate significant post-hoc comparisons using Dunnett's multiple comparisons test.

Table S4. Mean (M) and Standard Deviation (SD) of N1a Values Across Protocols and Time Points with Within-Protocol Modulation Results

Protocol		Baseline	Post 1	Post 2	Post 3	Post 4	Post 5	Post 6	F-value	p-value
Low-Frequency (*n* = 57)	M	-7.376	-8.309	-7.592	-7.543	-7.384	-7.551	-7.563	F (4.699. 241.2) = 1.448	.2111
	SD	4.197	5.302	4.448	4.171	4.365	4.029	4.513		
Repeated low-frequency (*n* = 33)	M	-7.607	-7.668	-6.56	-7.514	-7.385	-8.808	-7.772	F (4.567. 143.1) = 2.329	.0508
	SD	5.108	4.205	4.346	4.475	4.069	4.144	5.481		
High-frequency (*n* = 29)	M	-8.078	-9.2	-7.292	-8.804	-7.372	-7.625	-6.808	F (4.085. 113.7) = 3.215	.0147
	SD	4.201	4.696	4.341	4.958	3.838	4.08	4.065		
Theta-Pulse (*n* = 32)	M	-5.914	-6.529	-5.794	-6.479	-6.285	-6.715	-6.184	F (4.829. 149.7) = 0.7522	.5814
	SD	4.067	4.447	4.282	4.471	3.643	4.753	4.902		

Note. Row effects were analyzed using one-way ANOVA with repeated measures to compare each post time point against the baseline. A restricted maximum likelihood (REML) model was fitted when data were missing. Stars (*, **, ***, ****) indicate significant post-hoc comparisons using Dunnett's multiple comparisons test.

Table S5. Mean (M) and Standard Deviation (SD) of N1b Values Across Protocols and Time Points with Within-Protocol Modulation Results

Protocol		Baseline	Post 1	Post 2	Post 3	Post 4	Post 5	Post 6	F-value	p-value
Low-Frequency (*n* = 57)	M	-1.878	-4.243****	-3.401**	-3.144*	-2.689	-3.009*	-2.654	F (5.100. 247.3) = 5.889	<.0001
	SD	3.581	4.524	3.256	3.546	3.611	3.091	3.609		
Repeated low-frequency (*n* = 33)	M	-1.418	-4.287**	-2.505	-3.075	-2.627	-3.576**	-2.484	F (3.608. 113.0) = 5.130	.0012
	SD	4.057	3.426	3.146	4.015	2.826	3.431	3.636		
High-frequency (*n* = 29)	M	-2.48	-4.588**	-2.347	-3.823	-2.344	-3.327	-1.837	F (4.858. 136.0) = 5.689	.0001
	SD	3.402	3.554	4.286	3.262	3.42	3.51	4.34		
Theta-Pulse (*n* = 32)	M	-0.7766	-2.933*	-1.082	-2.361*	-1.908	-2.302	-1.537	F (5.067. 157.1) = 3.932	.0021
	SD	3.896	3.535	3.594	3.011	3.327	3.817	3.875		

Note. Row effects were analyzed using one-way ANOVA with repeated measures to compare each post time point against the baseline. A restricted maximum likelihood (REML) model was fitted when data were missing. Stars (*, **, ***, ****) indicate significant post-hoc comparisons using Dunnett's multiple comparisons test.

Table S6. Mean (M) and Standard Deviation (SD) of P2 Values Across Protocols and Time Points with Within-Protocol Modulation Results

Protocol		Baseline	Post 1	Post 2	Post 3	Post 4	Post 5	Post 6	F-value	p-value
Low-Frequency (*n* = 57)	M	1.395	2.06	0.941	1.73	2.059	2.197	2.383*	F (5.123. 271.5) = 2.758	.018
	SD	2.959	3.141	3.009	3.156	3.097	2.927	3.131		
Repeated low-frequency (*n* = 33)	M	1.999	2.378	1.395	1.234	1.298	0.817	2.19	F (5.013. 157.1) = 1.982	.0839
	SD	3.954	3.892	3.727	2.843	3.148	3.341	4.175		
High-frequency (*n* = 29)	M	1.067	1.4	2.639	0.7401	0.9628	1.565	2.295	F (4.682. 131.1) = 2.241	.0580
	SD	3.082	3.711	3.349	3.395	3.494	3.243	3.981		
Theta-Pulse (*n* = 32)	M	1.728	2.21	2.023	1.554	2.761	1.367	2.616	F (4.810. 149.1) = 1.889	.1023
	SD	3.593	3.604	3.099	3.082	3.704	3.317	3.793		

Note. Row effects were analyzed using one-way ANOVA with repeated measures to compare each post time point against the baseline. A restricted maximum likelihood (REML) model was fitted when data were missing. Stars (*, **, ***, ****) indicate significant post-hoc comparisons using Dunnett's multiple comparisons test.
